# Notes and News

**Published:** 1887-10-01

**Authors:** 


### NOTES AND NEWS.

COLLECTED AND COMMUNICATED.

THE contract for the new wing to be erected in connection with the Taunton and Somerset Hospital has been entered into, the tender of Mr. William Templeman, builder, of Taunton, amounting to £3,340, having been finally accepted.

AT Newcastle-on-Tyne the Hospital Sunday Fund has been in operation since 1870, or exactly seventeen years. During that time no less a sum than £55,350 19*s.* 2*d.* has been distributed to the various hospitals and charitable institutions of the town—a noble record of popular benevolence.

THE enlargement of the Blackburn Infirmary and the vastly increased number of out-patients treated make it desirable that an additional house-surgeon shall be employed. The committee, with commendable energy, have met and unanimously altered the rules so as to admit of the appointment of a second house-surgeon without delay.

SMALL-POX appears to be spreading at Sheffield, and especially in the neighbourhood of the borough hospital. An outcry is beginning to be heard that the hospital is a source of danger to the inhabitants in its vicinity. The town authorities should look into this question at once.

A DEMONSTRATION of 1,500 members of the different Trade Societies took place at Middlesbrough on Saturday. A procession, accompanied by several bands of music, marched to the Theatre Royal, which was crowded. The Mayor occupied the chair, and there were also on the stage Mr. Isaac Wilson, M.P., Mr. W. H. Patterson, secretary to the Durham Miners' Association, and a large number of members of the Town Council. A collection was taken at the close of the proceedings on behalf of the hospital.

A STEADY downpour of rain spoilt the Hospital Saturday collection at Eastbourne this year. But the managers, with true British acuteness, were determined not to be beaten by the weather. Finding that the skies continued unpropitious, and that everybody kept indoors, they publicly announced that Hospital Saturday was postponed, and that the usual collection would be taken on the 24th. This prudent foresight deserves an appropriate reward.

THE secretary to the Great Northern Central Hospital, Caledonian Road, N., writes, that on account of the large number of accidents which have lately been treated in this institution, the stock of old linen used for the dressings is quite exhausted ; and he appeals for gifts of this necessary article, and of old under-linen and men's clothes, which will be thankfully received by the matron.

THE cottage hospital to be erected at Coldstream will cost £1,000, and £350 more will be required in annual subscriptions. About £600 has been promised towards the building, and £150 in annual subscriptions has been announced. The Earl of Home opened a bazaar within the grounds of the castle, on an extensive scale, in aid of the building and endowment funds.

A SEA-SIDE home, erected at the sole cost of the Marchioness of Ailsa, has recently been opened at Calgean, near Maybole. N.B. The home is for the benefit of the working-classes. Anyone convalescing from a non-infectious disease may obtain admittance on application, provided he can pay the small sum per week which is charged. Lady Ailsa keeps the management entirely in her own hands, and is ably seconded by the matron, Mrs. Hay, A bowling-green and other means of amusement are important features of the new institution.

SOME ladies of Hastings, with true feminine goodwill, are uniting to help in furnishing the new hospital which is now completed. A few pounds spent in this way by every well-to-do inhabitant, under the guidance of the hospital officials, would not be felt seriously by the contributors of the money, and might relieve the hospital finances to the extent of some thousands of pounds. It is much to be desired that the generous example set by the few will be extensively followed in Hastings and the neighbourhood. The visitors, too, might very properly be asked to permit a small contribution to be levied from each of them. As the Scotch proverb says, "Mony a little maks a muckle."

A SUM of £2 10*s.* has been handed over to the Keighley Cottage Hospital, being the net proceeds of a concert given on behalf of the funds. The expenses amounted to £14 15*s.* Surely the plums in this hospital pudding were very deficient. Perhaps our informant is incorrect, and the £14 15*s.* may be the amount given to the hospital, whilst £2 10*s.* was spent in expenses. If this be so we shall be glad to be informed, but if otherwise, we venture to express the hope that next year, when a concert is given, the hospital may have the dog handed over to it instead of the tail.

THE managers of the Preston Infirmary open their building periodically for inspection by working people with a view to enlisting the interest of all classes. On Thursday evening about a hundred females, drafted from the different mills in the town and district, paid a visit to the institution, some of them having walked a distance of several miles for this purpose. The company assembled about seven o'clock, and were conducted through the wards in three divisions, headed by the matron, Mr. Saw, the chairman of the mills and workshops committee, Mr. Forest, one of the honorary secretaries, and Messrs. Gardner and Kershaw. Dr. Collinson, assisted by one of the nurses, devoted about twenty minutes to each set of visitors, and gave in that time some useful information about the treatment of patients at home, showing how much may be done with very simple appliances and by unskilled hands to promote personal comfort and alleviate suffering in all classes of homes. The visitors expressed themselves as exceedingly gratified by what they had seen. All large town hospitals might usefully follow Preston's example.

THE following vacancies are announced this week, the amount of the salary being placed in each case after the name of the institution :—

**Resident Medical Officers.**

Birmingham General Hospital. Assistant House Surgeon.
British Hospital, Buenos Ayres. Medical Officer.—£200, board.
East London Hospital for Children. Two Clinical Assistants.
Female Lock Hospital, Harrow Road. House Surgeon.—£100.
Halifax Infirmary and Dispensary. Assistant House Surgeon.—£50.

**Non-resident Medical Officers.**

Bristol Royal Infirmary. Obstetric Physician.
East Suffolk Hospital, Ipswich. Honorary Physician.
Great Northern Central Hospital. Obstetric Physician.

**Matrons.**

Newport Infirmary and Dispensary.—£50, with board, etc.
South-Western Fever Hospital, Stockwell.—£100, with board, etc.

**Trained Nurses.**

Hampstead Home Hospital. Several.—From £20.
Royal Albert Hospital, Devonport. One.—£25.
Royal Portsmouth Hospital. Head Nurse for Women's Wards.—£25.
South-Eastern Hospital, New Cross.—Several trained, £30 to £36.
South-Eastern Hospital, New Cross. Several Assistant.—£22 to £26.
Stroud Hospital. One. £23, with uniform.

---

A LONDON hospital nurse, writing to the *Exeter Western Times*, expresses deep sympathy with those inhabitants of the city who have shared in the sudden calamity which has recently fallen upon it. The resources of the local hospital were strained to the utmost to receive all those who needed its attention and care. The funds appear to be in a somewhat depressed condition, and the nurse thinks this a very proper time for making a special appeal to the more prosperous inhabitants. She herself offers to head a special fund by a subscription. The managers of the hospital will do well to take the hint, and bring before a sympathetic public the needs and uses of the institution.

---

WAKEFIELD has for some years had an open-air concert on behalf of the Clayton Hospital on a special Sunday. The result from a financial point of view has generally been all that could be desired. This year, in consequence of some objections to the Sunday performance, the concert took place on Saturday. It is very unfortunate that the change should have been made, so far as the hospital is concerned. The concert itself was good and the audience select, but the majority of the people of the town seem to have stayed away, and the proceeds compare very unfavourably with those of former years. The Wakefield Hospital managers should reconsider the question before next year.

---

IT is satisfactory to find that ninety-nine officers of the metropolitan police have passed a satisfactory examination in "First Aid to the Injured," and have received certificates of proficiency. Colonel Duncan, M.P., presided at a meeting for the distribution of certificates at Scotland Yard, and Mrs. Duncan presented them to the officers. Amongst those present were Sir Herbert Perrott, Bart., Deputy-Inspector John Coates, R.N., Dr. A. O. Mackellar, chief surgeon of the metropolitan police, Dr. Garrick Steel, chief medical officer to the G.P.O., Dr. Taylor, etc. Colonel Duncan said that years ago they could hardly have anticipated that the work of the Association would develop as it had. The great hold the hospitaller work had taken of the public was evidenced by the fact that in the last twelve months 5,000 more certificates were gained than had ever been gained in any previous year. The value of knowledge obtained in these classes was shown by their having heard that in the recent American railway collision many lives might have been saved by intelligent handling of the injured.

THE principal causes of the present depressed condition of hospital finances in London are, according to Mr. Ormond Hutt, the three following : (*a*) Too many large hospitals in close proximity ; (*b*) too many special hospitals ; (*c*) divided authority in the management. By way of remedy, Mr. Hutt would keep twenty general and ten special hospitals, fairly distributed over the metropolis, and let the rest go. He would "abolish secretaries and committees of management ; place each hospital in the sole charge of a medical man, with an efficient medical and surgical staff,' and over all would appoint "a Hospital Board, consisting of four commissioners, an eminent layman as chief commissioner, with a physician, surgeon, and chemist as assistant commissioners ; all to be appointed by the Home Secretary." For providing the wherewithal " the Hospital Sunday Fund should form the nucleus, the difference between expenditure and income being met by a rate and a grant from Parliament." There are several dead flies which spoil the odour of this pot of ointment.

---

MANY people dislike giving to charitable objects because they say so much of the money is spent on officials, administrative details, and in other ways which cannot be called charitable. It is much to be desired that money given for benevolent purposes should be subject to as few deductions as possible. As much as 10 per cent. is given by some hospitals to collectors, but there are means of obtaining money lying ready to hand which do not involve any deduction at all. Among these means church and chapel collections stand perhaps foremost. The extension scheme at Aberdeen is estimated to cost £30,000, and the managers desire that the whole of this sum shall be raised before the contracts are entered upon. Ministers of different denominations have been specially appealed to with a view to their organising house-to-house collections or congregational contributions, and it is pointed out that movements of this nature have in some quarters produced large sums. The parish of Strichen has sent £80 ; Ratheo, £20 ; New Pitsligo, £22 ; Slains parish church, £15 ; and Aberdour, £35. These are all small parishes, at least they all have very small populations. The whole sum produced up to the present time by this and other means amounts to £24,886. Hospital committees and secretaries should avail themselves of these inexpensive means of collection very much more largely than they do. It is eminently desirable that a general effort be made all over the country to bring parishes and congregations into immediate relationship with the hospital system, with a view to a vastly decreased cost in the collection of moneys for charitable purposes.

---

*Apropos* of the above, it may be mentioned that The Hospitals Association, under the presidency of Sir Andrew Clark, Bart., M.D., is about to make an energetic effort to interest and influence clergymen and ministers of religion, and all those of the public who take part in medical and other charities in this direction. Enlightened friends of hospitals should make a point of acquainting themselves with the nature and value of this scheme which the Association has in hand. If physicians and surgeons, secretaries and other officials, subscribers and friends of hospitals generally, would join hands with the Association in this great and important effort, very large results might be expected within a short time. Although so much money is collected annually on behalf of charities, it is really given by a very few persons, and there are vast areas of virgin soil ready for cultivation on hospital lines which no one even attempts to reach. A combined, intelligent, and earnest effort on the part of those whose business it is to promote the prosperity of hospitals could not but result in a great and surprising success.

Sir Walter Foster and others addressed crowded meetings of the members of the Foresters' Societies in the town-hall and Curzon Hall, Birmingham, on behalf of the fund for furnishing the convalescent home which those Societies have founded at Clent. Sir Walter said he was specially glad and proud of what they were doing in reference to this convalescent home. He had frequently seen men go out of the wards half restored, or because being the bread-winners of the families they could not wait until they had gained their full strength. Too often he had seen them return very soon broken down by work which they were not strong enough to perform, and condemned to suffer a second illness. This state of things was passing away, because the great towns were surrounded by such institutions as that which the order was about to found. By their efforts they would relieve the local hospitals, and thus benefit many of their poorer brothers. Liberal collections were made on behalf of the fund.

The clerk of the Luton Board of Guardians, who must be an exceedingly wise person, is reported to have said at a recent meeting of the Board that the cottage hospital seemed to be chiefly for the patients of the medical officers attached to the hospital. What possible objection there can be to a medical man sending a patient to the cottage hospital and attending him there himself, it is difficult to conceive. The moment the patient is within the hospital walls he ceases to be remunerative to the doctor, and therefore there can be no suspicion of mercenary motive in the matter. What motive, other than a good one, any medical man could have in taking such a course we find it impossible to imagine. Perhaps the enlightened clerk of the Luton Guardians' Board has more efficient visual organs.

The Hon. Mrs. Bulkeley Owen claims for the North Ormesby Cottage Hospital that it was the first institution of its kind established in this country. The immediate cause of its being founded was a melancholy catastrophe which occurred at the works of Messrs. Snowdon and Hopkins in the year 1838. A large stationary boiler burst with disastrous effect, severely injuring sixteen or seventeen men. But although the town of Middlesbrough at that time had a population of no less than 15,000 people, there was no infirmary or hospital nearer than Newcastle, some thirty miles away. Several of the men were sent there, and two died on the way; others were taken home, and two found a sick-room in the stable of an inn in Stockton Street. Another poor fellow, whose bed took fire under him in Dacre Street, was finally deposited, suffering agonies from frightful open wounds, upon a bundle of straw. The wretched condition of these poor creatures moved the sympathies of many kindly people in the town. Sister Mary Jaques, a native of Kirkleathen, who had got her training in the institution which sent Florence Nightingale out to her noble work, had for some time previous to this explosion been residing at Coatham, ready for any philanthropic labour that presented itself. She was sent for when the accident happened, and to the best of her means ministered to the wants of the poor sufferers. In consequence of the sympathy thus aroused a cottage hospital was immediately projected, and a house and two cottages were taken in Albert Road, and here Sister Mary and her two assistant nurses dwelt and carried on their work until the present hospital was built. This new departure at North Ormesby soon began to attract attention all over the country, and many places quickly followed the example thus set. At the present time every small town with the least pretensions to public spirit is not satisfied without the possession of an efficient cottage hospital.

There is reason to hope that the scarlet-fever epidemic has reached its height, and may now begin to abate. It is true the daily number of new cases has not appreciably diminished, but the deaths are fewer in proportion, and there seems to be no tendency to an increase. It is probable, too, that the parochial authorities are much more vigilant than they were a fortnight ago, and that the net being finer, many cases are brought to the hospitals which, under a less vigilant *régime*, would have been permitted to remain at home.

The first three volumes of *Misericordia*, the organ of the Guild of St. Barnabas for nurses, have been presented by the Rev. E. F. Russell, the chaplain, to the library of The Hospitals Association. It is hoped this example will be extensively followed. All works bearing on hospitals will be welcomed.

The Board of Management of the Norfolk and Norwich Hospital have appointed Mr. Albert E. Boyce secretary and house steward. There were 68 candidates. Mr. Boyce has for the last three years held the office of house-steward at the General Hospital, Birmingham.

"Tartar" is dead: a victim to his own rare and disinterested character. Tartar was an intelligent and philanthropic terrier, the friend and property of Mrs. Burt, of the "Eglinton Arms," Bristol. During the eight months preceding his death he collected no less a sum than £12 10*s.* for the Bristol Royal Infirmary. He had a collecting-box, which he regarded as his special charge, and with which he actively canvassed his mistress's customers for subscriptions for the good cause. Few could say "No" to Tartar. A *post mortem* showed that death had resulted from the presence of one or more pennies which he had swallowed in his eagerness to secure them for his box. Alas, poor Tartar! Who will raise a monument to his memory?

The Rev. J. W. Irvine appears to be one of the best friends the Colchester Hospital has. In anticipation of the Hospital Saturday collection to be taken on October 1st, a meeting of the committee has been held and much activity is displayed. At the meeting letters were read from a number of ladies who were unable to attend, but who promised to assist in the movement. Some of the ladies did not reply at all to the circulars sent out, and Mr. Irvine at once undertook to call upon them, in company with Mr. Bland, to endeavour to secure their co-operation. This kind of personal and active interest in the work cannot be too highly commended.

The editor of the *Eastern Daily Press* has published unfavourable statements with regard to the success of the bazaar in aid of the Lowestoft Hospital. Among other things, the *Press* states that "the weather proved unfavourable, and well-meant efforts ended in disaster." As against this, Mr. Frederick Morse, the treasurer to the hospital, says that the paragraph in the *Press* has been contradicted, but that the contradiction has not been published by the editor, and goes on to affirm that, "notwithstanding the most unfavourable weather, their efforts were crowned with complete success, and the fund raised far exceeds the most sanguine expectation." If Mr. Morse and his committee are satisfied, we do not see why the editor of the *Eastern Daily Press* should not also be pleased, and have the courtesy to say so.

